# Body composition and inflammation variables as the potential prognostic factors in epithelial ovarian cancer treated with Olaparib

**DOI:** 10.3389/fonc.2024.1359635

**Published:** 2024-04-25

**Authors:** Xingzi Guo, Jie Tang, Haifeng He, Lian Jian, Ouyang Qiang, Yongzhi Xie

**Affiliations:** ^1^ Department of Gynecologic Oncology, Hunan Cancer Hospital/The Affiliated Cancer Hospital of Xiangya School of Medicine, Central South University, Changsha, China; ^2^ Department of Radiology, The Third Xiangya Hospital, Central South University, Changsha, China; ^3^ Department of Radiology, Hunan Cancer Hospital/The Affiliated Cancer Hospital of Xiangya School of Medicine, Central South University, Changsha, China

**Keywords:** epithelial ovarian cancer, poly (ADP-ribose) polymerase inhibitors, body composition, inflammation variables, progression free survival

## Abstract

**Background:**

Epithelial ovarian cancer (EOC) is a significant cause of mortality among gynecological cancers. While Olaparib, a PARP inhibitor, has demonstrated efficacy in EOC maintenance therapy, individual responses vary. This study aims to assess the prognostic significance of body composition and systemic inflammation markers in EOC patients undergoing initial Olaparib treatment.

**Methods:**

A retrospective analysis was conducted on 133 EOC patients initiating Olaparib therapy. Progression-free survival (PFS) was assessed through Kaplan-Meier analysis and Cox proportional hazards regression. Pre-treatment computed tomography images were utilized to evaluate body composition parameters including subcutaneous adipose tissue index (SATI), visceral adipose tissue index (VATI), skeletal muscle area index (SMI), and body mineral density (BMD). Inflammatory markers, such as neutrophil-to-lymphocyte ratio (NLR), platelet-to-lymphocyte ratio (PLR), serum albumin, and hemoglobin levels, were also measured.

**Results:**

The median follow-up duration was 16 months (range: 5-49 months). Survival analysis indicated that high SATI, high VATI, high SMI, high BMD, low NLR, and low PLR were associated with decreased risk of disease progression (all p < 0.05). Multivariate analysis identified several factors independently associated with poor PFS, including second or further lines of therapy (HR = 2.16; 95% CI = 1.09-4.27, p = 0.027), low VATI (HR = 3.79; 95% CI = 1.48-9.70, p = 0.005), low SMI (HR = 2.52; 95% CI = 1.11-5.72, p = 0.027), low BMD (HR = 2.36; 95% CI = 1.22-4.54, p = 0.010), and high NLR (HR = 0.31; 95% CI = 0.14-0.69, p = 0.004). Subgroup analysis in serous adenocarcinoma patients revealed distinct prognostic capabilities of SATI, VATI, SMI, PLR, and NLR

**Conclusion:**

Body composition and inflammation variables hold promise as predictors of therapeutic response to Olaparib in EOC patients. Understanding their prognostic significance could facilitate tailored treatment strategies, potentially improving patient outcomes.

## Introduction

Ovarian cancer ranked as the third most prevalent gynecological cancer in the global cancer statistics of 2020. The worldwide incidence of new cases reached 313,959, with 207,252 resulting in fatalities (1). In China, the statistics for 2022 reported 57,090 new cases and 24,494 deaths (2), which demonstrate only a slight decline compared to the 2015 data (3). The high mortality rate can be attributed to the advanced stage at the time of ovarian cancer diagnosis (4). For decades, the conventional treatment approach for ovarian cancer has been radical debulking surgery followed by platinum-based combination chemotherapy, which has proven to be the most effective and widely used method (5). However, within five years, approximately 70% of patients experience recurrence (6). The efficacy of subsequent lines of chemotherapy diminishes with each relapse, resulting in a minority of advanced-stage ovarian cancer patients surviving for five years with traditional treatment (7).

The synthetic lethal approach of targeting the DNA repair pathway is the mechanism of Poly (ADP-ribose) polymerase (PARP) inhibitors as maintenance therapy in ovarian cancers (8). With increasing evidence supporting the use of maintenance therapy, Olaparib has become popular due to its longer progression-free survival (PFS) and overall survival (OS) in the SOLO1 trial (9). This trial treated patients with BRCA1/2 mutation diagnosed with high-grade serous/endometrioid ovarian cancer with Olaparib, which resulted in a 70% lower risk of disease progression or death. In the PAOLA-1 trial, Olaparib treatment for homologous recombination deficient (HRD) tended to extend the PFS and OS (10). There is also strong evidence that relapsed platinum-sensitive-ovarian cancer responds well to maintenance drugs such as Olaparib (11–13). Undoubtedly, the PFS and OS are the reliable terms of predictive treatment outcomes, who receive PARP inhibitors. However, not every patient benefits from Olaparib as maintenance therapy, and the outcomes of PARP inhibitors for the specific patients cannot be determined until progression. Therefore, the reliable and validated biomarkers from patients are needed to predict their response to these drugs.

Abdominal adipose tissue, especially the distributions of visceral adipose tissue (VAT)and subcutaneous adipose tissue (SAT) measured by quantitative computer tomography (QCT), have been acknowledged as a good prognostic biomarker for PFS and OS after surgery, radiation, or classical chemotherapy (14, 15). Overweight has been identified as a high-risk factor for several cancers (16, 17), such as prostate, breast and colorectal cancers. Emerging evidence also suggests that sarcopenic obesity, characterized by severe obesity and low skeletal muscle area (SMA), might be a predictor of cancer (18). Many observational studies have shown that sarcopenic obesity as the biomarker predicts a poor OS in cancer patients (19), as well as the loss of body mineral density (BMD) (20). Furthermore, research has focused on the body composition as a predictor of response and toxicity to cancer immune checkpoint inhibitors (21). Meanwhile, the efficacy of apatinib as vascular endothelial growth factor (VEGF)-targeted therapy in predicting the outcome of ovarian cancer patients by evaluating the distinct adipose tissue has been reported (22).

In addition to the patient’s body composition, systemic inflammation is believed to play an important role in the progression of ovarian cancers (23). Inflammation-based prognostic indicators, such as neutrophil-to-lymphocyte ratio (NLR) (24) and the platelet-to-lymphocyte ratio (PLR) (25), have been reported in various cancers. The level of hemoglobin and serum albumin can also reflect nutritional status, which has been investigated as a prognostic factor in cancers (26).

We aimed to explore whether CT-based body composition (VAT, SAT, SMA, and BMD), systemic inflammation (NLR and PLR), and nutritional status could serve as prognostic predictors for epithelial ovarian cancer (EOC) patients treated with Olaparib.

## Methods

### Patients

In this retrospective analysis, we examined patients diagnosed with Stage IIB-IV EOC as classified by the International Federation of Gynecology and Obstetrics (27). These individuals exhibited either BRCA1/2 mutations (germline and/or somatic mutations) and/or were identified as HRD-positive. Following optimal debulking surgery, they underwent first-line platinum-based chemotherapy. Subsequently, they received an initial treatment with Olaparib (300 mg bid) at our institution between November 2018 and December 2021. The maximum duration of Olaparib maintenance therapy extended to 2 years, with no instances of treatment discontinuation attributed to side effects. Discontinuation events were solely linked to early cessation prompted by disease progression. For individuals undergoing Olaparib maintenance therapy for epithelial ovarian cancer, common side effects, including nausea, fatigue, anemia, thrombocytopenia, insomnia, leucopenia, constipation, diarrhea, and joint pain, were typically mild to moderate (grades 1-3). Notably, bone marrow suppression, such as anemia, platelet reduction, and leucopenia, often fell within this range. Additionally, other side effects were generally of grade 1 severity. Additionally, patients with platinum-sensitive, relapsed epithelial ovarian cancer who had received 2 or more lines of treatment initially treated with Olaparib were also included. The inclusion criteria were as follows: (a) individuals who had undergone a diagnostically acceptable abdominal CT within 1 month before initiating Olaparib treatment; (b) those with histologically confirmed EOC. The exclusion criteria were as follows: (1) incomplete clinical follow-up data; (2) poor quality CT scans; (3) absence of routine hematological and biochemical examinations within 7 days before the initial Olaparib treatment; (4) combined with bevacizumab as maintenance therapy; (5) individuals receiving steroids or other immunomodulatory agents within 1 month prior to starting Olaparib treatment or those diagnosed with infections or immunodeficiencies. PFS was defined as the time (in months) from the initiation of Olaparib treatment to disease progression or the last follow-up in December 2022.

Clinical and pathological data, including age, weight, height, tumor grading and histology type, lines of treatment, pre-treatment complete blood counts (neutrophil, lymphocyte, and platelet counts), serum albumin, and hemoglobin, were extracted from retrospective medical records at the time of Olaparib initiation and before administering the first dose (300 mg bid). NLR was calculated by dividing the absolute neutrophil count by the absolute lymphocyte count, and PLR was calculated by dividing the absolute platelet count by the absolute lymphocyte count. Serum hemoglobin increased ≥110 g/L was defined as normal, and a serum albumin <40 g/l was defined as hypoalbuminemia. Height and weight measurements acquired within 14 days before the treatment. Body mass index (BMI) was calculated using the formula weight/height2 (kilograms per square meter). Patients were classified into four weight categories: underweight (BMI < 18.5 kg/m^2^), normal weight (18.5 kg/m^2^ ≤ BMI ≤ 22.9 kg/m^2^), overweight (23 kg/m^2^ ≤ BMI ≤ 24.9 kg/m^2^), and obese (BMI ≥ 25 kg/m^2^).

### CT analysis

**Figure 1:**
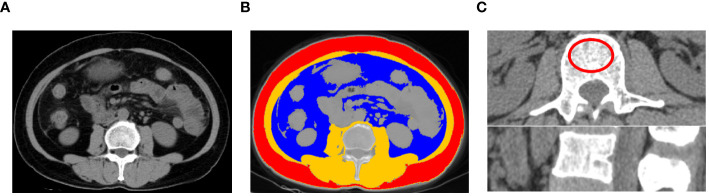
An example of segmentation of body composition. **(A)** original image; **(B)** Subcutaneous adipose tissue (red), visceral adipose tissue (blue), and skeletal muscle area (Brown) from an axial image at the level of L3 vertebra of a CT scan; **(C)** Measurement of bone mineral density of L2 vertebra a CT scan.

Abdominal CT images were obtained before initiating Olaparib treatment (within a month). CT examinations were performed in the axial plane with 5-mm-thick sections using a 64-row CT scanner (Somatom definition AS large-aperture, Siemens Healthcare, Germany) and a 256-row CT scanner (revolution, GE Healthcare, USA). A single slice of each patient’s baseline CT image was selected at the third lumbar vertebra (L3) as the standard for assessing body composition. The segmentation of SAT, VAT and SMA were performed by using 3D Slicer software (version 4.11.2; Boston, MA, USA) (**Figure 1A**) and the area of interest were manually calculated. The threshold for adipose tissue was set between -190 and -30 Hounsfield units (HU) (SAT: ranging from -190 to -30 HU; VAT: ranging from -150 to -50 HU). SMA was measured within the range of -29 to +150 HU (18) (**Figure 1B**). The cross-sectional area values were normalized for height, and the measurements were labeled as SATI, VATI, SMI following previously published methods [(cm^2^)/(m^2^)] (28). Additionally, BMD values were calculated at the L2 vertebra level and the area of the interest was approximately 4 cm^2^ (29) (**Figure 1C**).

### Statistical analyses

R software (Version 4.2.3) was used to perform all data analyses. Continuous variables were expressed as mean ± standard error. Categorical variables were compared using the chi-square test. The optimal cutoff value for continuous variables (including NLR, PLR, VATI, SATI, SMI, and BMD) was determined using the surv_cutpoint function based on the previously published methods (30, 31). Kaplan-Meier survival curves and log-rank tests were conducted using the “survival” and “survminer” R packages to illustrate the survival differences between the two groups. To identify potential independent prognostic factors, univariate analyses were performed initially, and a multivariate Cox proportional hazards regression (stepwise model) analysis was subsequently conducted, including all variables with a p-value less than 0.05 from the univariate analysis. To reduce the potential confounding and selection bias, propensity score matching (PSM) analysis was carried out and 1:1 nearest-neighbor matching. Propensity scores were calculated using logistic regression models with the clinical, body composition and inflammation variables. Statistical significance was defined as *p* < 0.05.

## Results

### Patients characteristics

**Figure 2:**
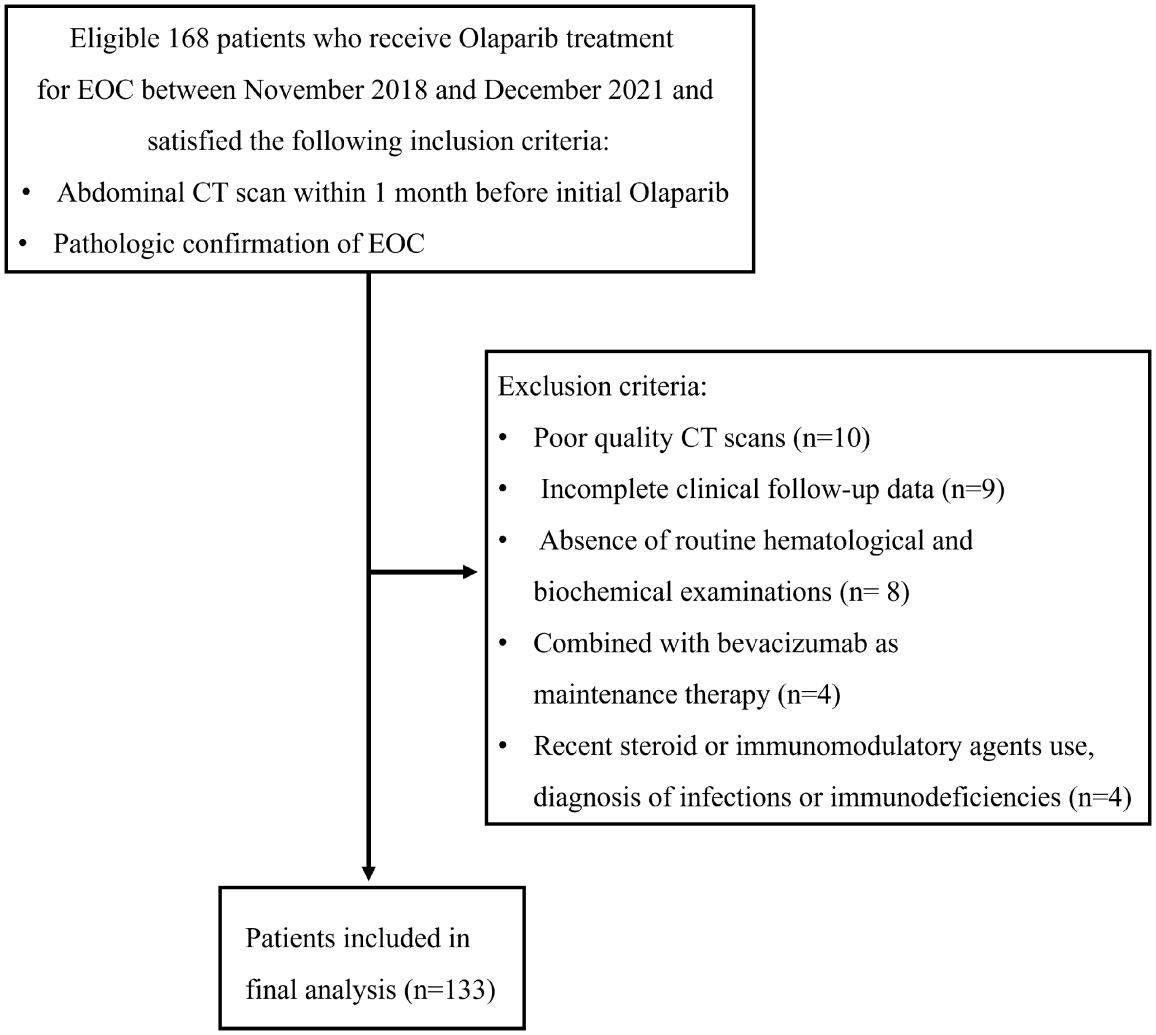
Flow diagram depicting patient selection process.

**Figure 3:**
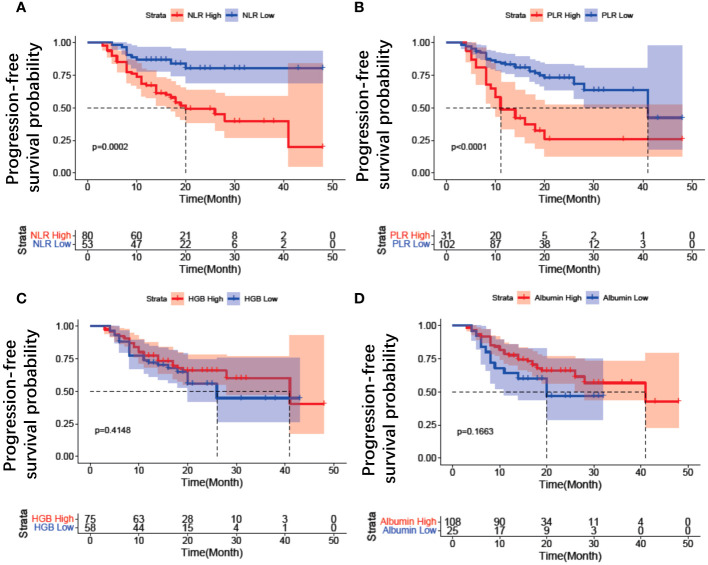
Kaplan–Meier estimates of progression free survival for inflammation variables in patients with EOC treated with Olaparib. **(A)** NLR change, **(B)** PLR change, **(C)** HGB change, **(D)** Albumin change. NLR, neutrophil-to-lymphocyte ratio; PLR, platelet-to-lymphocyte ratio; HGB, hemoglobin.

Between November 2018 and December 2021, a total of 168 patients underwent screening, of whom 35 patients were excluded (**Figure 2**). Ultimately, 133 patients were included in this study, with a mean age of 54.32 ± 8.29 years (range: 28-71), mean serum albumin of 43.10 ± 3.61 g/l, mean hemoglobin of 111.25 ± 15.30 g/L, mean NLR of 2.69 ± 1.82, and mean PLR of 159.75 ± 91.82. The median follow-up duration was 16 months (range: 5-49 months). Serous adenocarcinoma was the most common subtype, accounting for 84.9% (113/133) of the total patients. Fifty-seven out of 133 (42.8%) patients received first-line treatment. The clinical characteristics of the patients are summarized in **Table 1**. The optimal cut-off values for NLR, PLR, determined using the surv_cutpoint R function, were 2.11, and 192, respectively. To facilitate further analysis, patients were categorized into high or low groups based on these cut-off values (NLR ≤ 2.11 and > 2.11; PLR ≤ 192 and > 192). Kaplan-Meier curve analysis for PFS demonstrated clear differentiation between the two groups for NLR and PLR (both *p*<0.001), indicating a significant association between decreased NLR, decreased PLR, and favorable PFS (**Figures 3A, B**). However, serum hemoglobin and albumin were not significantly associated with PFS (**Figures 3C, D**).

**Table 1:** Patient characteristics: Demographics.

Parameters	N
Age (year), mean ± SD	54.32 ± 8.29
≥60	32
<60	101
BMI (kg/m2), mean ± SD	23.23 ± 2.94
BMI range*	
Underweight (<18.5)	7
Normal (18.5–22.9)	57
Overweight (23.0–24.9)	35
Obesity (≥25.0)	34
Histology types	
Serous	113
Endometroid	2
Clear cell	2
Mucinous	1
Others	15
Tumor grading	
Well-Moderate differentiated	10
Low differentiated	123
Number of previous chemotherapy lines	
1 line	57
2-3 lines	67
>3 lines	9
FIGO staging	
I-II	18
III-IV	115
Laboratory Tests	
Neutrophil count (10^9^/L)	3.15 ± 1.69
Lymphocyte count (10^9^/L)	1.34 ± 0.53
Platelet count (10^9^/L)	180.61 ± 72.60
NLR	2.69 ± 1.82
PLR	159.75 ± 91.82
Hemoglobin (g/L)	111.25 ± 15.30
Albumin (g/L)	43.10 ± 3.61
Body Composition Parameters, mean ± SD	
SATI (cm^2^/m^2^)	61.33 ± 18.95
VATI (cm^2^/m^2^)	28.17 ± 14.24
SMI (cm^2^/m^2^)	41.20 ± 5.93
BMD (HU)	153.96 ± 49.31

FIGO, The International Federation of Gynecology and Obstetrics; BMI, body mass index; SATI, subcutaneous adipose tissue index; VATI, visceral adipose tissue index; SMI, skeletal muscle area index; BMD, bone mineral density; NLR, neutrophil-to-lymphocyte ratio; PLR, platelet-to-lymphocyte; SD, standard deviation.

### Body composition and serum inflammation factors associated with progression-free survival

**Figure 4:**
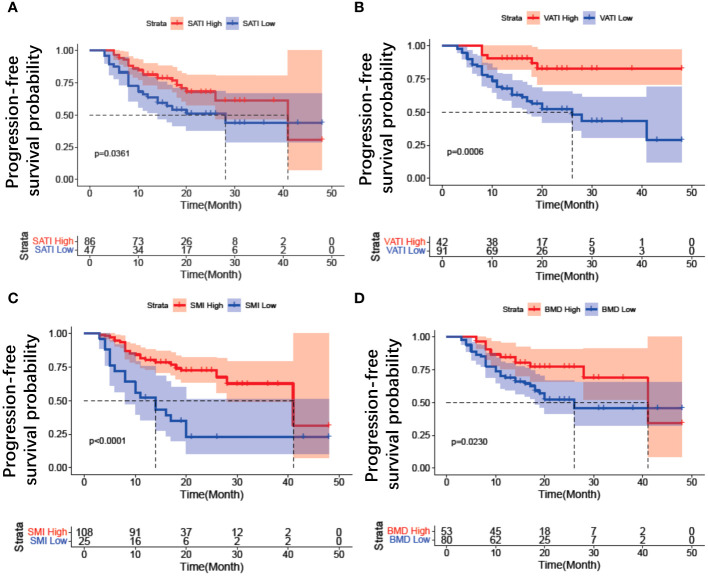
Kaplan–Meier estimates of progression free survival for body composition in patients with EOC treated with Olaparib. **(A)** SATI change; **(B)** VATI change; **(C)** SMI change; **(D)** BMD change. SATI, subcutaneous adipose tissue index; VATI, visceral adipose tissue index; SMI, skeletal muscle area index; BMD, bone mineral density.

High intra-observer consistency was observed for the measurement of SAT, VAT, SM, and BMD, with pretreatment intraclass association coefficients of 0.906, 0.873, 0.864, 0.836, respectively. After normalizing for height, the average values for subcutaneous adipose tissue index (SATI), visceral adipose tissue index (VATI), and skeletal muscle area index (SMI) were 61.33 ± 18.95, 28.17 ± 14.24, and 41.20 ± 5.93 (cm²)/(m²), respectively (**Table 1**). Patients were divided into high or low groups based on cut-off values of 50.7 cm²/m² for SATI, 35.7 cm²/m² for VATI, 37.0 cm²/m² for SMI, and 163 HU for BMD (**Table 2**). The risk of disease progression in the high group was further analyzed. Kaplan-Meier curve analysis revealed that patients with high SATI (**Figure 4A**), high VATI (**Figure 4B**), high SMI (**Figure 4C**), and high BMD (**Figure 4D**) had a lower risk of disease progression compared to those with low SATI (*p* = 0.036), low VATI (*p* = 0.0006), low SMI (*p* < 0.001), and low BMD (*p* = 0.023), respectively. SMI was the strongest prognostic factor for disease progression.

**Table 2:** Cox proportional hazard models for progression-free survival of patients with epithelial ovarian cancer during Olaparib maintenance treatment.

	Univariable analysis		Multivariable analysis	
	HR (95% CI)	P value	HR (95% CI)	P value
Age (<60 years)	0.87 (0.45-1.67)	0.668		
BMI (<23 kg/m2)	1.31 (0.74-2.32)	0.351		
FIGO staging (I-II)	0.85 (0.36-2.01)	0.716		
Histology type (non-Serous)	0.76 (0.37-1.58)	0.455		
Tumor grading (low differentiated)	1.22 (0.48-3.10)	0.671		
Chemotherapy lines (second or further lines)	2.19 (1.16-4.14)	0.016	2.16 (1.09-4.27)	0.027
NLR (<2.11)	0.28 (0.13- 0.58)	<0.001	0.31 (0.14-0.69)	0.004
PLR (<192)	0.28 (0.16- 0.50)	<0.001	0.50 (0.25-1.00)	0.050
HGB (<110 g/L)	1.27 (0.72-2.25)	0.40		
Albumin (<40 g/L)	1.59 (0.82-3.05)	0.17		
SATI (<50.7 cm2/m2)	1.82 (1.03-3.22)	0.038	0.60 (0.28-1.29)	0.190
VATI (<35.7 cm2/m2)	2.61 (1.22-5.58)	0.013	3.79 (1.48-9.70)	0.005
SMI (<37.0 cm2/m2)	3.34 (1.85-6.02)	<0.001	2.52 (1.11-5.72)	0.027
BMD (<163 HU)	2.06 (1.09-3.89)	0.027	2.36 (1.22-4.54)	0.010

BMI, body mass index; FIGO, The International Federation of Gynecology and Obstetrics; NLR, neutrophil-to-lymphocyte ratio; PLR, platelet-to-lymphocyte ratio; HGB, hemoglobin; SATI, subcutaneous adipose tissue index; VATI, visceral adipose tissue index; SMI, skeletal muscle area index; BMD, bone mineral density; HR, hazard ratio; CI confidence interval.

Based on PSM analysis, we obtained matched patients for SATI, VATI, SMI, BMD, NLR, and PLR variables respectively at 1:1 ratio. We then performed the survival analysis to evaluate prognosis outcomes. Kaplan-Meier curve of SATI, VATI, SMI, BMD, NLR, and PLR could clearly distinguish two groups (high vs low) (all p < 0.05), consistent with previous results of whole patients (**Supplementary Figure 1**).

Univariable Cox proportional hazard analysis was conducted to assess the association between clinical parameters (including tumor grading, histology type, chemotherapy lines, body composition and serum inflammation factors) and progression-free survival in patients. NLR, PLR and SMI were found to be the strongest prognostic parameter for progression-free survival (*p* < 0.001) (**Table 2**). Second or further lines therapy, high SATI, high VATI, and high BMD were associated with decreased progression-free survival compared to the corresponding group (*p* < 0.05) (**Table 2**). Multivariable Cox proportional hazard models for progression-free survival were also presented in **Table 2**. In the multivariate analysis, chemotherapy lines, three body composition parameters and one serum inflammation factor were identified as independent factors associated with poor PFS: second or further lines (HR = 2.16; 95% CI = 1.09-4.27, *p* = 0.027), low VATI (HR = 3.79; 95% CI = 1.48-9.70, *p* = 0.005), low SMI (HR = 2.52; 95% CI = 1.11-5.72, *p* = 0.027), low BMD (HR = 2.36; 95% CI = 1.22-4.54, *p* = 0.010), and high NLR (HR = 0.31; 95% CI = 0.14-0.69, *p* = 0.004).

**Figure 5:**
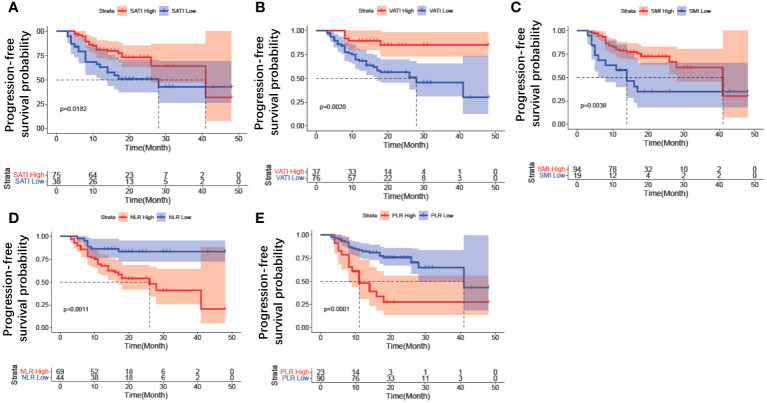
Kaplan–Meier curve analysis of clinical parameters for patients with serous adenocarcinoma. **(A)** SATI change; **(B)** VATI change; **(C)** SMI change; **(D)** NLR change; **(E)** PLR change. SATI, subcutaneous adipose tissue index; VATI, visceral adipose tissue index; SMI, skeletal muscle area index; NLR, neutrophil-to-lymphocyte ratio; PLR, platelet-to-lymphocyte ratio.

To remove the difference of histological subtype in the results, Kaplan-Meier curve analysis was further performed only for the population with serous adenocarcinoma. The results revealed that patients with high SATI (*p* = 0.0182) (**Figure 5A**), high VATI (*p* = 0.002) (**Figure 5B**), high SMI (*p* = 0.0038) (**Figure 5C**), low NLR (*p* = 0.001) (**Figure 5D**), and low PLR (*p*<0.0001) (**Figure 5E**) had a lower risk of disease progression. The Kaplan-Meier curves of other clinical parameters, including chemotherapy lines and BMD, could not distinguish two groups. Furthermore, we analyzed the variables between patients with first line maintenance or relapse maintenance. There were no differences in body composition and inflammation variables between these two groups (**Supplementary Table 1**).

## Discussion

PARP enzymes are expressed in various metabolic tissues and organs, including skeletal muscle, endocrine glands, and adipose tissue (32). It is plausible that PARP plays a role in facilitating DNA repair in adipocytes, thus improving metabolic imbalances associated with obesity (33). Moreover, studies have reported that PARP inhibitors can enhance skeletal muscle function by promoting mitochondrial biogenesis and protecting against diet-induced obesity (34). Notably, Olaparib, one of the PARP inhibitors, can also influence adipocyte formation (35). PARP inhibitors are closely associated with the metabolism of tissues such as muscle and fat. Numerous studies have shown that assessing body composition through imaging techniques can predict the efficacy of drugs in cancer treatment. In this context, our study aims to elucidate the effectiveness of Olaparib in patients with EOC.

In this study, we investigated the use of Olaparib as a maintenance drug for epithelial ovarian cancer patients who had BRCA mutations or HRD positive as the first-line therapy and experienced platinum-sensitive recurrence. Advanced epithelial ovarian cancer (AEOC) is a heterogeneous disease (36) with varying responses to Olaparib. Our study is the first to demonstrate the clinical significance of body composition and serum inflammatory indexes in predicting the outcomes of patients treated with Olaparib. We found that the adipose tissue index, skeletal muscle mass index, and bone density measured by QCT were associated with the prognosis of EOC patients treated with Olaparib. Univariate and multivariable logistic regression analyses revealed that decreased VATI, SMI, and BMD were independent predictors of poor progression-free survival.

Accumulating evidence suggests that visceral adipose tissue not only functions as an energy storage organ but also plays a role in tumor development (37). Several studies have demonstrated an association between adipose tissue and various types of cancers. In some cases, lower visceral adipose tissue has been linked to the development of gastrointestinal cancer and head and neck squamous cell carcinoma (38, 39), which aligns with our findings. However, higher VAT values have been associated with worse outcomes in metastatic colorectal cancer (40). Moreover, clinically observable indicators like adipose tissue could serve as reliable markers for evaluating the efficacy of targeted drugs. For instance, in AEOC patients treated with anti-angiogenic therapy such as bevacizumab, adipose tissue levels were significantly associated with overall survival (41). Similarly, adipose tissue has been identified as a predictor of the efficacy of VEGF receptor inhibitors in ovarian cancer (22). These findings support the hypothesis that adipose tissue could be a potential predictor of clinical drug outcomes.

Muscle mass and bone density are also reliable indicators of functional status and biomarkers of treatment outcomes (42). Lower skeletal muscle index has been shown to predict reduced overall survival in AEOC patients undergoing primary debulking surgery and in melanoma patients treated with immune checkpoint inhibitors (43). Additionally, a lower skeletal muscle index, as determined by CT scans, has been identified as a predictor of poor overall survival prognosis in small-cell lung cancer (44) and as a marker for shorter time to tumor progression in metastatic breast cancer (45). BMD, assessed before treatment, is independent prognostic factors for OS in patients with advanced cholangiocellular adenocarcinoma (46). The loss of BMD has been linked to shorter overall survival in AEOC patients undergoing primary debulking surgery and adjuvant chemotherapy, corroborating our study findings (20).

Additionally, the relationship between cancer-related inflammation response and alterations in muscle wastage and visceral adipose tissue is increasingly recognized. Inflammation markers, notably the NLR, have emerged as potential prognostic indicators for sarcopenia. The integration of NLR with other markers might enhance prognostic precision (47). Inflammation is now acknowledged as a pivotal factor in the development of various cancers and is recognized as a hallmark of cancer (48). For patients undergoing chemotherapy, normalization of elevated NLR levels early in treatment may correlate with improved outcomes (49, 50). A NLR exceeding the defined threshold has been linked with a higher hazard ratio for survival outcomes in colorectal carcinoma, gastroesophageal carcinoma, non–small cell lung cancer, and renal cell carcinoma (51). Moreover, a heightened NLR value correlates with an immunosuppressive profile (52) and portends a poorer overall survival rate in ovarian cancer patients. It is important to underscore that the malfunctioning of immune cells, particularly macrophages residing in adipose tissue, leading to chronic inflammation, has been intricately linked to the progression of cancer. Elevated baseline NLR has also been associated with poor survival in patients treated with immunotherapy, including those with cancer cachexia (53, 54). From this, one might deduce that high NLR concentrations can influence both muscle atrophy and visceral adipose tissue dynamics. The inhibition of PARP has demonstrated efficacy in moderating the inflammatory response, subsequently enhancing survival in sepsis scenarios (55). To a certain degree, Olaparib might mitigate inflammation, thus augmenting survival, although such a postulation warrants further empirical and foundational research validation. In our research, we discerned an association between NLR-a systemic inflammation-based prognostic marker-and the efficacy of Olaparib in EOC patients. Elevated NLR was pinpointed as an independent prognostic determinant of adverse PFS during Olaparib administration. Analogous observations have been noted in ovarian cancer studies, where inflammation markers such as elevated NLR and PLR correlate with advanced tumor staging, metastasis, and platinum resistance (25). Similarly, the elevated PLR is expected to have poor prognosis in non-small cell lung cancer (56) and hepatocellular cancer (57).

### Limitations and future directions

Our study faces limitations. Firstly, its retrospective nature impedes acquiring dynamic CT evaluation and inflammatory index data, hindering understanding of temporal changes in body composition, and inflammatory markers during Olaparib maintenance therapy. Secondly, exclusively including Asian individuals limits generalizability due to potential genetic variations. Thirdly, small sample size, single-center design, and potential selection bias raise concerns about broader applicability. These underscore the need for cautious interpretation and emphasize future prospective, multi-center studies with diverse populations.

To validate findings and explore mechanisms, several future research directions are warranted. Firstly, prospective studies or trials with larger, diverse populations are essential to verify prognostic significance of body composition and inflammation variables in EOC patients treated with Olaparib. Incorporating longitudinal assessments to track changes in these markers and their correlation with treatment response is crucial.

Secondly, mechanistic studies are needed to elucidate biological pathways influencing treatment outcomes. Exploring the role of immune cells, particularly adipose tissue-resident macrophages, in modulating tumor microenvironment and response to PARP inhibition could offer insights into novel treatment strategies.

## Conclusions

In conclusion, the early identification of patients displaying diminished VATI, SMI, and BMD, coupled with elevated NLR, provides preliminary evidence suggestive of an increased risk in disease progression and offers insights for guiding therapeutic interventions. These observations may hold significant clinical implications, particularly in tailoring personalized treatment approaches for EOC patients undergoing Olaparib maintenance. Our study serves as a preliminary step, highlighting the need for continued exploration and comprehensive investigations in this intricate clinical context.

## Data availability statement

The raw data supporting the conclusions of this article will be made available by the authors, without undue reservation.

## Ethics statement

The studies involving humans were approved by Ethics Committee of Hunan Cancer Hospital. The studies were conducted in accordance with the local legislation and institutional requirements. The participants provided their written informed consent to participate in this study. Written informed consent was obtained from the individual(s) for the publication of any potentially identifiable images or data included in this article.

## Author contributions

XG: Writing – original draft, Writing – review & editing, Project administration. JT: Data curation, Formal analysis, Writing – review & editing. HH: Data curation, Methodology, Writing – review & editing. LJ: Investigation, Resources, Software, Writing – review & editing. OQ: Investigation, Resources, Writing – review & editing. YX: Supervision, Validation, Writing – review & editing, Data curation.
