# Supplementary material for: Body composition and inflammation variables as the potential prognostic factors in epithelial ovarian cancer treated with Olaparib
Source: Front Oncol. 2024 Apr 25;14:1359635. doi: 10.3389/fonc.2024.1359635 (PMC11079183; doi:10.3389/fonc.2024.1359635)
Supplement: Supplementary file 1 [file DataSheet_1.pdf]

Supplementary Table 1 Chi-square test result

Variables	Total (n = 133)	First line treatment (n=57)	Second or further lines treatment (n=76)	Statistic	P
Age, n (%)				χ^2=3.734	0.053
< 60 years	101 (75.94)	48 (84.21)	53 (69.74)		
≥60 years	32 (24.06)	9 (15.79)	23 (30.26)		
BMI, n (%)				χ^2=2.109	0.146
< 23 kg/m2	65 (48.87)	32 (56.14)	33 (43.42)		
≥23 kg/m2	68 (51.13)	25 (43.86)	43 (56.58)		
FIGO stage, n (%)				χ^2=0.771	0.380
I-II	18 (13.53)	6 (10.53)	12 (15.79)		
III-IV	115 (86.47)	51 (89.47)	64 (84.21)		
NLR, n (%)				χ^2=1.383	0.240
<2.11	53 (39.85)	26 (45.61)	27 (35.53)		
≥2.11	80 (60.15)	31 (54.39)	49 (64.47)		
PLR, n (%)				χ^2=0.014	0.906
<192	102 (76.69)	44 (77.19)	58 (76.32)		
≥192	31 (23.31)	13 (22.81)	18 (23.68)		
HGB, n (%)				χ^2=0.431	0.512
<110 g/L	58 (43.61)	23 (40.35)	35 (46.05)		
≥110 g/L	75 (56.39)	34 (59.65)	41 (53.95)		
Albumin, n (%)				χ^2=1.051	0.305
<40 g/L	25 (18.80)	13 (22.81)	12 (15.79)		
≥40 g/L	108 (81.20)	44 (77.19)	64 (84.21)		
SATI, n (%)				χ^2=0.175	0.675
<50.7 cm^2/m^2	47 (35.34)	19 (33.33)	28 (36.84)		
≥50.7 cm^2/m^2	86 (64.66)	38 (66.67)	48 (63.16)		
VATI, n (%)				χ^2=2.274	0.132
<35.7 cm^2/m^2	91 (68.42)	43 (75.44)	48 (63.16)		
≥35.7 cm^2/m^2	42 (31.58)	14 (24.56)	28 (36.84)		
SMI, n (%)				χ^2=1.482	0.223
<37.0 cm^2/m^2	25 (18.80)	8 (14.04)	17 (22.37)		
≥37.0 cm^2/m^2	108 (81.20)	49 (85.97)	59 (77.63)		
BMD, n (%)				χ^2=1.383	0.240
<163 HU	80 (60.15)	31 (54.39)	49 (64.47)		
≥163 HU	53 (39.85)	26 (45.61)	27 (35.53)		

Abbreviations: FIGO, The International Federation of Gynecology and Obstetrics; BMI, body mass index; SATI, subcutaneous adipose tissue index; VATI, visceral adipose tissue index; SMI, skeletal muscle area index; BMD, bone mineral density; NLR, neutrophil-to-lymphocyte ratio; PLR, platelet-to-lymphocyte; SD, standard deviation.

Note: Chi-Square Test ($p < 0.05$), Data presented are numbers of patients, with percentages in parentheses.
