# Supplementary material for: Body composition and inflammation variables as the potential prognostic factors in epithelial ovarian cancer treated with Olaparib
Source: Front Oncol. 2024 Apr 25;14:1359635. doi: 10.3389/fonc.2024.1359635 (PMC11079183; doi:10.3389/fonc.2024.1359635)
Supplement: Supplementary file 2 [file DataSheet_2.pdf]

Supplementary Figure 1

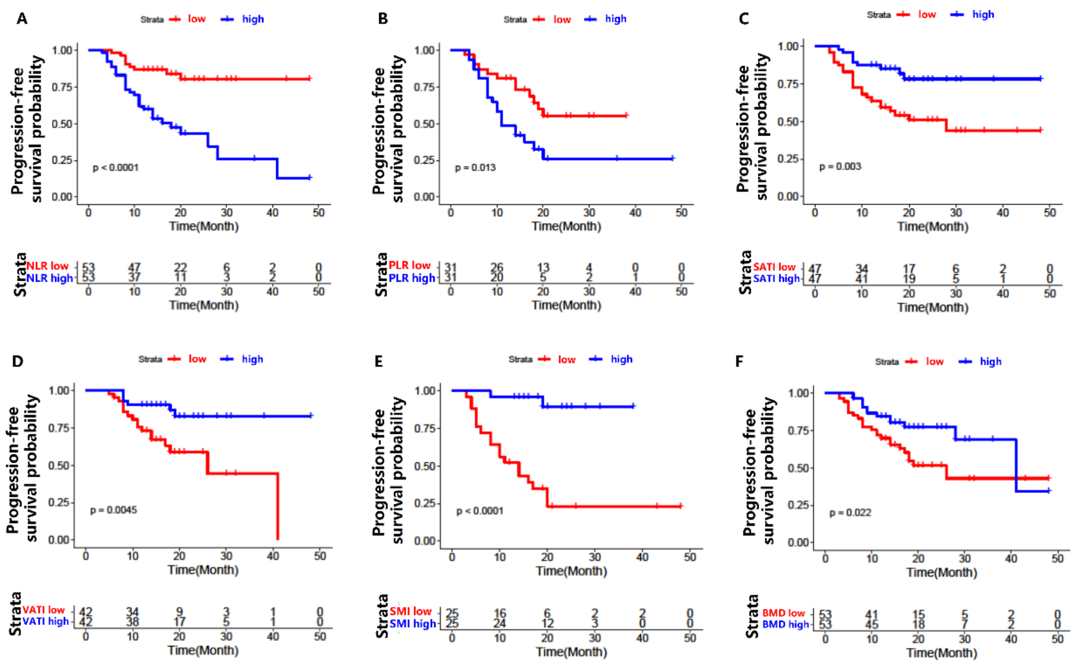

Survival curves using Kaplan–Meier analysis after propensity score matching. Kaplan–Meier estimates of progression free survival for body composition in patients with EOC treated with Olaparib. A: NLR change; B: PLR change; C: SATI change; D: VATI change; E: SMI change; F: BMD change. NLR, neutrophil-to-lymphocyte ratio; PLR, platelet-to-lymphocyte ratio; SATI, subcutaneous adipose tissue index; VATI, visceral adipose tissue index; SMI, skeletal muscle area index; BMD, bone mineral density.
